# Poly[tetra­deca­aqua­tetra­kis­(μ_3_-imidazole-4,5-dicarboxyl­ato)hexa-μ_3_-sulfato-cobalt(II)hexa­samarium(III)]

**DOI:** 10.1107/S1600536811030169

**Published:** 2011-07-30

**Authors:** Li-Cai Zhu, Si-Ming Zhu

**Affiliations:** a School of Chemistry and the Environment, South China Normal University, Guangzhou 510631, People’s Republic of China; b School of Light Industry and Food Science, South China University of Technology, Guangzhou 510641, People’s Republic of China

## Abstract

In the title three-dimensional compound, [CoSm_6_(C_5_H_2_N_2_O_4_)_4_(SO_4_)_6_(H_2_O)_14_]_*n*_, the Co^II^ ion is six-coordinated with two O atoms and two N atoms from two imidazole-4,5-dicarboxyl­ate ligands and two coordinated water mol­ecules, giving a slightly distorted octa­hedral geometry. One Sm^III^ ion is eight-coordinated in a bicapped trigonal–prismatic coordination geometry by four O atoms from two imidazole-4,5-dicarboxyl­ate ligands, two O atoms from two SO_4_^2−^ anions and two coordinated water mol­ecules. The other two Sm^III^ ions are nine-coordinated in a tricapped trigonal–prismatic coordination geometry; one of these Sm^III^ ions is bonded to four O atoms from two imidazole-4,5-dicarboxyl­ate ligands, three O atoms from three SO_4_^2−^ anions and two water O atoms, and the other Sm^III^ ion is coordinated by one O atom and one N atom from one imidazole-4,5-dicarboxyl­ate ligand, five O atoms from three SO_4_^2−^ anions, as well as two coordinated water mol­ecules. The crystal structure is further stabilized by N—H⋯O, O—H⋯O, and C—H⋯O hydrogen-bonding inter­actions.

## Related literature

For the application of lanthanide transition metal heterometallic complexes with bridging multifunctional organic ligands, see: Cheng *et al.* (2006 ▶); Kuang *et al.* (2007 ▶); Sun & Yang (2007 ▶); Zhu *et al.* (2010 ▶).
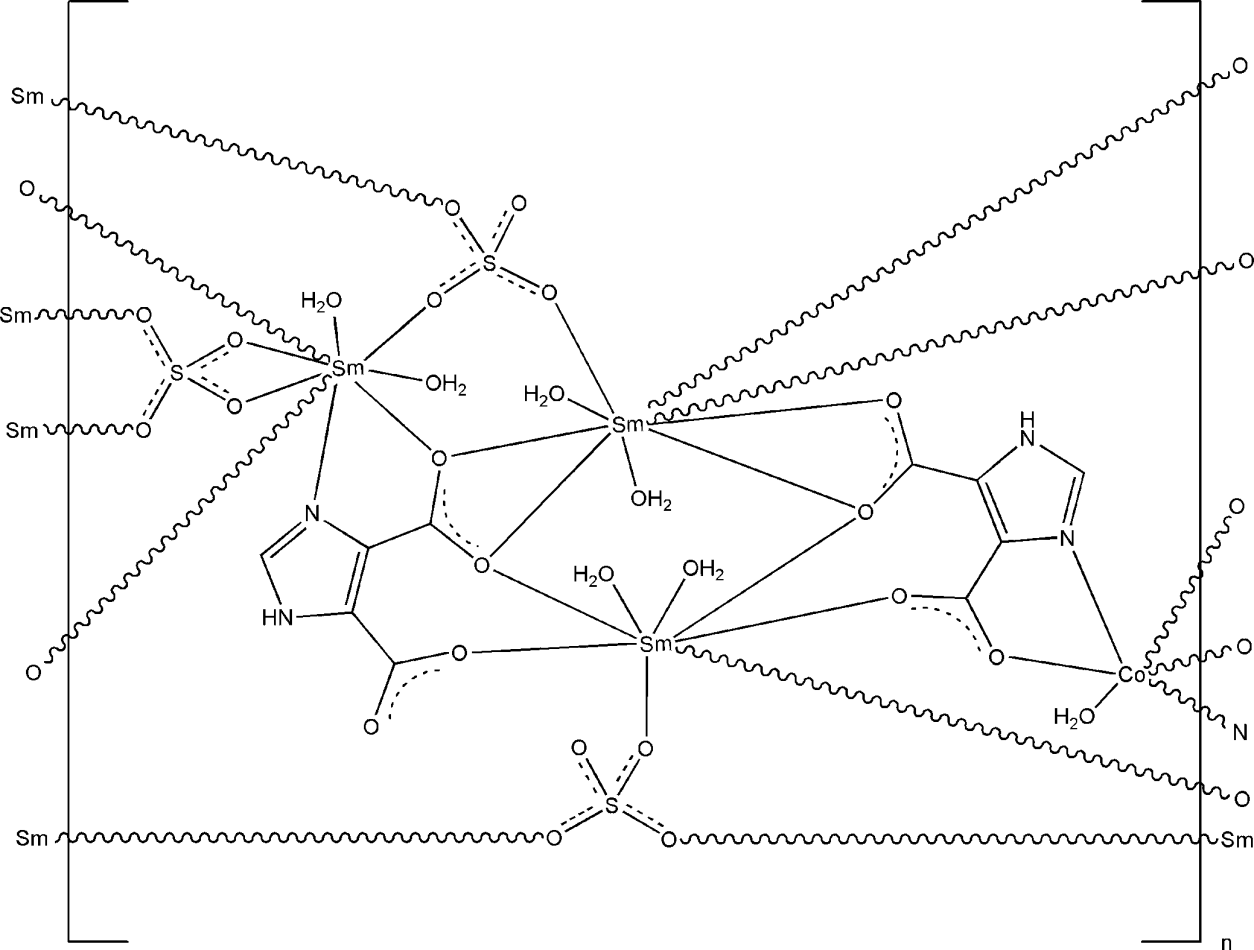


## Experimental

### 

#### Crystal data

[CoSm_6_(C_5_H_2_N_2_O_4_)_4_(SO_4_)_6_(H_2_O)_14_]
*M* *_r_* = 2406.08
Triclinic, 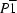

*a* = 6.6395 (10) Å
*b* = 9.5090 (14) Å
*c* = 21.619 (3) Å
α = 97.068 (2)°
β = 94.391 (2)°
γ = 98.006 (2)°
*V* = 1335.2 (3) Å^3^
*Z* = 1
Mo *K*α radiation
μ = 7.17 mm^−1^
*T* = 296 K
0.20 × 0.18 × 0.15 mm

#### Data collection

Bruker APEXII area-detector diffractometer
Absorption correction: multi-scan (*SADABS*; Sheldrick, 1996 ▶) *T*_min_ = 0.260, *T*_max_ = 0.341
6967 measured reflections
4716 independent reflections
3971 reflections with *I* > 2σ(*I*)
*R*_int_ = 0.024

#### Refinement

*R*[*F* ^2^ > 2σ(*F* ^2^)] = 0.031
*wR*(*F* ^2^) = 0.070
*S* = 1.02
4716 reflections
478 parameters
23 restraints
H atoms treated by a mixture of independent and constrained refinement
Δρ_max_ = 1.00 e Å^−3^
Δρ_min_ = −1.03 e Å^−3^

### 

Data collection: *APEX2* (Bruker, 2004 ▶); cell refinement: *SAINT* (Bruker, 2004 ▶); data reduction: *SAINT*; program(s) used to solve structure: *SHELXS97* (Sheldrick, 2008 ▶); program(s) used to refine structure: *SHELXL97* (Sheldrick, 2008 ▶); molecular graphics: *XP* in *SHELXTL* (Sheldrick, 2008 ▶); software used to prepare material for publication: *SHELXL97* (Sheldrick, 2008 ▶).

## Supplementary Material

Crystal structure: contains datablock(s) I, global. DOI: 10.1107/S1600536811030169/om2450sup1.cif

Structure factors: contains datablock(s) I. DOI: 10.1107/S1600536811030169/om2450Isup2.hkl

Additional supplementary materials:  crystallographic information; 3D view; checkCIF report

## Figures and Tables

**Table 1:** Hydrogen-bond geometry (Å, °)

*D*—H⋯*A*	*D*—H	H⋯*A*	*D*⋯*A*	*D*—H⋯*A*
N2—H1⋯O18^i^	0.87 (6)	2.06 (5)	2.916 (8)	170 (6)
O1*W*—H1*W*⋯O7	0.82 (5)	2.52 (8)	3.140 (8)	133 (7)
O1*W*—H1*W*⋯O16^i^	0.82 (5)	2.12 (6)	2.777 (6)	136 (8)
N4—H2⋯O7^ii^	0.87 (4)	1.94 (5)	2.803 (8)	171 (8)
O1*W*—H2*W*⋯O1^iii^	0.82 (5)	2.51 (6)	3.247 (7)	150 (7)
O2*W*—H3*W*⋯O3*W*	0.83 (5)	2.02 (5)	2.809 (8)	160 (6)
O2*W*—H4*W*⋯O6^iv^	0.83 (4)	2.55 (4)	3.303 (9)	152 (8)
O2*W*—H4*W*⋯O8^iv^	0.83 (4)	2.53 (7)	3.204 (8)	140 (6)
O3*W*—H5*W*⋯O4^v^	0.83 (4)	1.98 (5)	2.789 (7)	166 (8)
O3*W*—H6*W*⋯O1^iv^	0.83 (5)	1.93 (5)	2.732 (7)	161 (5)
O4*W*—H7*W*⋯O17	0.81 (5)	2.59 (6)	3.270 (8)	142 (7)
O4*W*—H7*W*⋯O19	0.81 (5)	2.20 (6)	2.874 (7)	141 (5)
O4*W*—H8*W*⋯O6^vi^	0.81 (7)	2.47 (7)	3.186 (8)	149 (6)
O5*W*—H10*W*⋯O7*W*^vii^	0.82 (6)	2.10 (6)	2.794 (8)	142 (6)
O6*W*—H11*W*⋯O14^viii^	0.81 (4)	1.99 (5)	2.751 (7)	157 (5)
O6*W*—H12*W*⋯O5*W*^viii^	0.81 (6)	2.27 (7)	3.053 (8)	162 (7)
O7*W*—H13*W*⋯O11^vii^	0.81 (5)	2.45 (5)	3.063 (6)	133 (7)
O7*W*—H13*W*⋯O12^vii^	0.81 (5)	2.06 (6)	2.855 (7)	168 (7)
O7*W*—H14*W*⋯O12^ix^	0.81 (5)	1.85 (5)	2.661 (7)	174 (7)
C5—H5⋯O3^x^	0.93	2.52	3.388 (8)	155
C10—H11⋯O17^ix^	0.93	2.54	3.430 (9)	161

Symmetry codes: (i) 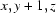; (ii) 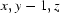; (iii) 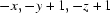; (iv) 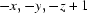; (v) 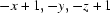; (vi) 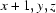; (vii) 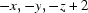; (viii) 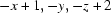; (ix) 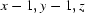; (x) 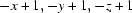.

**Fig. 1.**
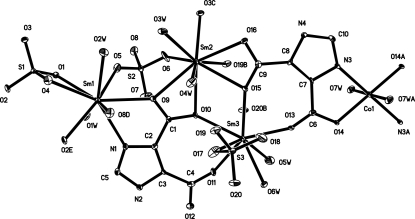
The molecular structure showing the atomic-numbering scheme and displacement ellipsoids drawn at the 30% probability level. Hydrogen atoms were omitted for clarity. Symmetry codes: (A) -x, -1 - y, 2 - z; (B) -1 + x, y, z; (C) - x, - y, 1 - z; (D) 1 + x, y, z; (E) 1 - x, 1 - y, 1 - z.

**Fig. 2.**
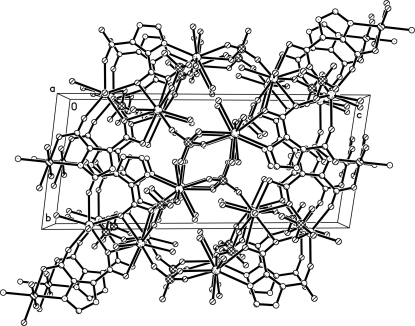
A view of the three-dimensional structure of the title compound. Hydrogen atoms were omitted for clarity.

## supplementary crystallographic information

### Comment

In the past few years, lanthanide-transition metal heterometallic complexs with bridging multifunctionnal organic ligands gained increasing interest, not only because of their impressive topological structures, but also due to their versatile applications in ion exchange, magnetism, bimetallic catalysis and as luminescent probes (Cheng *et al.*, 2006; Kuang *et al.*, 2007; Sun & Yang, 2007; Zhu *et al.*, 2010). As an extension of this research, the structure of the title compound, a new heterometallic coordination polymer has been determined which is presented in this artcle.

The asymmetric unit of the title compound (Fig. 1), contains a Co^II^ ion, three Sm^III^ ions, two imidazole-4, 5-dicarboxylate ligands, three SO_4_^2-^ anions, and seven coordinated water molecules. The Co^II^ ion lie in the inversion center and is six-coordinated with two O atoms from two coordinated water molecules, two O atoms and two N atoms from two imidazole-4, 5-dicarboxylate ligands, giving a slightly distorted octahedral geometry. The Sm^III^ ions exhibit three types of coordination environment. One Sm^III^ ion is eight-coordinated in a bicapped trigonal prismatic coordination geometry by four O atoms from two imidazole-4,5-dicarboxylate ligands, two O atoms from two SO_4_^2-^ anions and two coordinated water molecules. The other Sm^III^ ions are nine-coordinated in a tricapped trigonal prismatic coordination geometry; one of these Sm^III^ ions is bonded to four O atoms from two imidazole-4,5-dicarboxylate ligands, three O atoms from three SO_4_^2-^ anions and two water O atoms and the other Sm^III^ ion is coordinated by one O atom and one N atom from one imidazole-4, 5-dicarboxylate ligand, five O atoms from three SO_4_^2-^ anions as well as two coordinated water molecules. These metal coordination units are connected by bridging imidazole-4, 5-dicarboxylate and sulfate ligands, generating a three-dimensional network (Fig. 2). The crystal structure is further stabilized by N—H···O, O—H···O, and C—H···O hydrogen-bonding interactions between water molecules, SO_4_^2-^ anions, and imidazole-4, 5-dicarboxylate ligands (Table 1).

### Experimental

A mixture of CoSO_4_.7H_2_O(0.141 g, 0.5 mmol), Sm_2_O_3_(0.087 g, 0.25 mmol), imidazole-4,5-dicarboxylic acid (0.156 g, 1 mmol), and H_2_O(10 ml) was sealed in a 20 ml Teflon-lined reaction vessel at 443 K for 5 days then slowly cooled to room temperature. The product was collected by filtration, washed with water and air-dried. Red block crystals suitable for X-ray analysis were obtained.

### Refinement

H atoms bonded to C atoms were positioned geometrically and refined as riding, with C—H = 0.93 Å and *U*_iso_(H) = 1.2 *U*_eq_(C). H atoms bonded to N atoms and H atoms of water molecules were found from difference Fourier maps and refined isotropically with a restraint of N—H = 0.87 Å, O—H = 0.82 Å and *U*_iso_(H) = 1.5 *U*_eq_(N, O).

### Crystal data

**Table:**

[CoSm_6_(C_5_H_2_N_2_O_4_)_4_(SO_4_)_6_(H_2_O)_14_]	*Z* = 1
*M**_r_* = 2406.08	*F*(000) = 1139
Triclinic, *P*1	*D*_x_ = 2.992 Mg m^−^^3^
Hall symbol: -P 1	Mo *K*α radiation, λ = 0.71073 Å
*a* = 6.6395 (10) Å	Cell parameters from 2636 reflections
*b* = 9.5090 (14) Å	θ = 2.2–27.9°
*c* = 21.619 (3) Å	µ = 7.17 mm^−^^1^
α = 97.068 (2)°	*T* = 296 K
β = 94.391 (2)°	Block, red
γ = 98.006 (2)°	0.20 × 0.18 × 0.15 mm
*V* = 1335.2 (3) Å^3^	

### Data collection

**Table:**

Bruker APEXII area-detector diffractometer	4716 independent reflections
Radiation source: fine-focus sealed tube	3971 reflections with *I* > 2σ(*I*)
graphite	*R*_int_ = 0.024
φ and ω scan	θ_max_ = 25.2°, θ_min_ = 1.9°
Absorption correction: multi-scan (*SADABS*; Sheldrick, 1996)	*h* = −7→5
*T*_min_ = 0.260, *T*_max_ = 0.341	*k* = −11→11
6967 measured reflections	*l* = −25→25

### Refinement

**Table:**

Refinement on *F*^2^	Primary atom site location: structure-invariant direct methods
Least-squares matrix: full	Secondary atom site location: difference Fourier map
*R*[*F*^2^ > 2σ(*F*^2^)] = 0.031	Hydrogen site location: inferred from neighbouring sites
*wR*(*F*^2^) = 0.070	H atoms treated by a mixture of independent and constrained refinement
*S* = 1.02	*w* = 1/[σ^2^(*F*_o_^2^) + (0.0293*P*)^2^ + 2.3668*P*] where *P* = (*F*_o_^2^ + 2*F*_c_^2^)/3
4716 reflections	(Δ/σ)_max_ = 0.002
478 parameters	Δρ_max_ = 1.00 e Å^−^^3^
23 restraints	Δρ_min_ = −1.03 e Å^−^^3^

### Special details

**Table:**

Geometry. All e.s.d.'s (except the e.s.d. in the dihedral angle between two l.s. planes) are estimated using the full covariance matrix. The cell e.s.d.'s are taken into account individually in the estimation of e.s.d.'s in distances, angles and torsion angles; correlations between e.s.d.'s in cell parameters are only used when they are defined by crystal symmetry. An approximate (isotropic) treatment of cell e.s.d.'s is used for estimating e.s.d.'s involving l.s. planes.
Refinement. Refinement of *F*^2^ against ALL reflections. The weighted *R*-factor *wR* and goodness of fit *S* are based on *F*^2^, conventional *R*-factors *R* are based on *F*, with *F* set to zero for negative *F*^2^. The threshold expression of *F*^2^ > σ(*F*^2^) is used only for calculating *R*-factors(gt) *etc*. and is not relevant to the choice of reflections for refinement. *R*-factors based on *F*^2^ are statistically about twice as large as those based on *F*, and *R*- factors based on ALL data will be even larger.

### Fractional atomic coordinates and isotropic or equivalent isotropic displacement parameters (Å^2^)

**Table:**

	*x*	*y*	*z*	*U*_iso_*/*U*_eq_	
Sm3	0.33206 (6)	0.01643 (4)	0.860687 (16)	0.01396 (10)	
Sm2	0.06536 (5)	−0.11526 (4)	0.677756 (16)	0.01039 (9)	
Sm1	0.34787 (5)	0.29252 (4)	0.577813 (16)	0.01209 (10)	
Co1	0.0000	−0.5000	1.0000	0.0144 (3)	
S1	0.3305 (3)	0.35636 (18)	0.43568 (8)	0.0117 (3)	
S2	−0.1527 (3)	0.18219 (18)	0.62306 (8)	0.0144 (4)	
S3	0.7700 (3)	−0.05896 (19)	0.81404 (8)	0.0163 (4)	
N1	0.4459 (9)	0.4015 (6)	0.6919 (3)	0.0158 (13)	
N2	0.5267 (9)	0.5408 (6)	0.7811 (3)	0.0145 (13)	
N3	−0.0134 (9)	−0.5242 (6)	0.8997 (3)	0.0139 (13)	
N4	−0.0377 (9)	−0.5503 (6)	0.7965 (2)	0.0129 (12)	
C1	0.3005 (10)	0.1744 (7)	0.7196 (3)	0.0110 (14)	
C2	0.4007 (10)	0.3228 (7)	0.7395 (3)	0.0123 (14)	
C3	0.4487 (10)	0.4087 (7)	0.7958 (3)	0.0113 (14)	
C4	0.4360 (10)	0.3875 (7)	0.8624 (3)	0.0147 (15)	
C5	0.5213 (11)	0.5311 (8)	0.7190 (3)	0.0176 (16)	
H5	0.5655	0.6069	0.6975	0.021*	
C6	0.1796 (11)	−0.2840 (7)	0.9305 (3)	0.0154 (15)	
C7	0.0741 (10)	−0.3984 (7)	0.8801 (3)	0.0131 (15)	
C8	0.0601 (10)	−0.4121 (7)	0.8155 (3)	0.0114 (14)	
C9	0.1121 (11)	−0.3170 (7)	0.7687 (3)	0.0148 (15)	
C10	−0.0769 (10)	−0.6128 (7)	0.8472 (3)	0.0144 (15)	
H11	−0.1408	−0.7067	0.8459	0.017*	
O1	0.1806 (7)	0.3703 (5)	0.4827 (2)	0.0177 (11)	
O2	0.4117 (8)	0.4971 (5)	0.4209 (2)	0.0221 (12)	
O3	0.2273 (7)	0.2689 (5)	0.3784 (2)	0.0198 (11)	
O4	0.4904 (7)	0.2855 (5)	0.4640 (2)	0.0232 (12)	
O5	−0.0086 (8)	0.2036 (6)	0.5754 (2)	0.0259 (12)	
O6	−0.1534 (8)	0.0358 (5)	0.6398 (3)	0.0319 (14)	
O7	−0.0966 (9)	0.2882 (6)	0.6773 (3)	0.0410 (16)	
O8	−0.3591 (7)	0.1881 (5)	0.5942 (2)	0.0242 (12)	
O9	0.2794 (7)	0.1296 (5)	0.6617 (2)	0.0152 (10)	
O10	0.2340 (7)	0.0918 (5)	0.7580 (2)	0.0137 (10)	
O11	0.3918 (8)	0.2647 (5)	0.8764 (2)	0.0231 (12)	
O12	0.4709 (8)	0.4987 (5)	0.9016 (2)	0.0226 (12)	
O13	0.2724 (8)	−0.1722 (5)	0.9173 (2)	0.0205 (12)	
O14	0.1701 (8)	−0.3116 (5)	0.9862 (2)	0.0184 (11)	
O15	0.2062 (7)	−0.1901 (5)	0.7844 (2)	0.0148 (11)	
O16	0.0501 (8)	−0.3614 (5)	0.7124 (2)	0.0198 (11)	
O17	0.6400 (8)	0.0535 (6)	0.8074 (3)	0.0347 (14)	
O18	0.6559 (11)	−0.1679 (6)	0.8443 (3)	0.052 (2)	
O19	0.8096 (7)	−0.1215 (5)	0.7507 (2)	0.0212 (12)	
O20	0.9627 (7)	0.0049 (5)	0.8501 (2)	0.0243 (12)	
H1	0.563 (12)	0.623 (5)	0.804 (3)	0.036*	
H2	−0.042 (12)	−0.598 (7)	0.7595 (18)	0.036*	
O1W	0.1566 (8)	0.5002 (6)	0.6016 (2)	0.0261 (13)	
H1W	0.096 (10)	0.495 (9)	0.6332 (19)	0.039*	
H2W	0.065 (8)	0.500 (9)	0.574 (2)	0.039*	
O2W	0.2776 (10)	0.0581 (6)	0.5120 (3)	0.0341 (14)	
H3W	0.218 (13)	−0.017 (5)	0.522 (3)	0.051*	
H4W	0.292 (14)	0.032 (8)	0.4750 (16)	0.051*	
O3W	0.1551 (8)	−0.1832 (5)	0.5707 (2)	0.0227 (12)	
H5W	0.266 (5)	−0.213 (7)	0.567 (4)	0.034*	
H6W	0.073 (7)	−0.249 (6)	0.550 (3)	0.034*	
O4W	0.4309 (8)	−0.1323 (6)	0.6751 (2)	0.0259 (12)	
H7W	0.496 (10)	−0.127 (9)	0.7089 (18)	0.039*	
H8W	0.504 (10)	−0.087 (8)	0.654 (3)	0.039*	
O5W	0.1776 (9)	0.0980 (6)	0.9654 (3)	0.0349 (14)	
H9W	0.060 (6)	0.083 (9)	0.949 (4)	0.052*	
H10W	0.184 (12)	0.170 (6)	0.991 (3)	0.052*	
O6W	0.6080 (9)	0.0697 (5)	0.9447 (2)	0.0259 (13)	
H11W	0.651 (12)	0.152 (3)	0.959 (3)	0.039*	
H12W	0.639 (12)	0.021 (6)	0.971 (3)	0.039*	
O7W	−0.2592 (8)	−0.3945 (6)	0.9992 (2)	0.0225 (12)	
H13W	−0.311 (10)	−0.413 (8)	1.0306 (17)	0.034*	
H14W	−0.348 (8)	−0.425 (8)	0.971 (2)	0.034*	

### Atomic displacement parameters (Å^2^)

**Table:**

	*U*^11^	*U*^22^	*U*^33^	*U*^12^	*U*^13^	*U*^23^
Sm3	0.0175 (2)	0.01187 (19)	0.01055 (18)	−0.00508 (15)	−0.00139 (15)	0.00379 (14)
Sm2	0.01014 (19)	0.01094 (18)	0.00963 (18)	−0.00008 (14)	0.00035 (14)	0.00181 (13)
Sm1	0.0119 (2)	0.01235 (19)	0.01166 (18)	0.00027 (14)	0.00082 (14)	0.00227 (14)
Co1	0.0199 (8)	0.0113 (7)	0.0108 (7)	−0.0047 (6)	0.0021 (6)	0.0040 (5)
S1	0.0102 (9)	0.0135 (8)	0.0101 (8)	−0.0031 (7)	0.0000 (7)	0.0024 (6)
S2	0.0132 (9)	0.0142 (9)	0.0161 (9)	0.0018 (7)	0.0027 (7)	0.0034 (7)
S3	0.0127 (9)	0.0206 (9)	0.0133 (9)	−0.0015 (7)	0.0020 (7)	−0.0023 (7)
N1	0.022 (3)	0.015 (3)	0.011 (3)	−0.001 (3)	0.005 (3)	0.003 (2)
N2	0.018 (3)	0.011 (3)	0.013 (3)	−0.001 (3)	0.003 (3)	0.000 (2)
N3	0.015 (3)	0.011 (3)	0.014 (3)	−0.007 (2)	−0.001 (3)	0.004 (2)
N4	0.021 (3)	0.011 (3)	0.007 (3)	0.001 (2)	0.004 (3)	−0.002 (2)
C1	0.011 (4)	0.011 (3)	0.013 (3)	0.002 (3)	0.004 (3)	0.002 (3)
C2	0.008 (3)	0.016 (4)	0.013 (3)	0.003 (3)	0.002 (3)	0.001 (3)
C3	0.016 (4)	0.008 (3)	0.010 (3)	−0.003 (3)	0.006 (3)	−0.001 (3)
C4	0.011 (4)	0.015 (4)	0.017 (4)	0.006 (3)	−0.002 (3)	−0.004 (3)
C5	0.020 (4)	0.020 (4)	0.013 (4)	0.001 (3)	0.003 (3)	0.006 (3)
C6	0.015 (4)	0.018 (4)	0.011 (3)	0.001 (3)	0.001 (3)	−0.001 (3)
C7	0.015 (4)	0.009 (3)	0.016 (4)	0.000 (3)	0.003 (3)	0.009 (3)
C8	0.011 (4)	0.016 (4)	0.007 (3)	0.005 (3)	−0.002 (3)	0.003 (3)
C9	0.016 (4)	0.015 (4)	0.016 (4)	0.005 (3)	0.005 (3)	0.003 (3)
C10	0.009 (4)	0.013 (3)	0.021 (4)	−0.004 (3)	0.004 (3)	0.002 (3)
O1	0.015 (3)	0.022 (3)	0.013 (2)	−0.004 (2)	0.003 (2)	0.002 (2)
O2	0.030 (3)	0.016 (3)	0.020 (3)	−0.006 (2)	0.007 (2)	0.007 (2)
O3	0.019 (3)	0.024 (3)	0.013 (2)	−0.007 (2)	0.004 (2)	−0.004 (2)
O4	0.016 (3)	0.028 (3)	0.028 (3)	0.008 (2)	0.000 (2)	0.009 (2)
O5	0.015 (3)	0.040 (3)	0.023 (3)	−0.001 (2)	0.004 (2)	0.010 (2)
O6	0.015 (3)	0.018 (3)	0.066 (4)	0.000 (2)	0.002 (3)	0.025 (3)
O7	0.044 (4)	0.045 (4)	0.026 (3)	0.003 (3)	0.001 (3)	−0.021 (3)
O8	0.015 (3)	0.033 (3)	0.028 (3)	0.011 (2)	−0.001 (2)	0.010 (2)
O9	0.021 (3)	0.012 (2)	0.011 (2)	0.000 (2)	0.001 (2)	0.0008 (19)
O10	0.016 (3)	0.013 (2)	0.013 (2)	−0.002 (2)	0.005 (2)	0.0036 (19)
O11	0.041 (3)	0.012 (3)	0.016 (3)	−0.001 (2)	0.005 (2)	0.006 (2)
O12	0.031 (3)	0.017 (3)	0.017 (3)	−0.001 (2)	0.000 (2)	−0.003 (2)
O13	0.030 (3)	0.016 (3)	0.010 (2)	−0.011 (2)	−0.004 (2)	0.001 (2)
O14	0.028 (3)	0.013 (2)	0.010 (2)	−0.012 (2)	−0.001 (2)	0.0035 (19)
O15	0.021 (3)	0.010 (2)	0.012 (2)	−0.003 (2)	0.002 (2)	0.0015 (19)
O16	0.032 (3)	0.015 (3)	0.010 (3)	0.004 (2)	−0.004 (2)	−0.001 (2)
O17	0.022 (3)	0.059 (4)	0.026 (3)	0.022 (3)	0.003 (3)	−0.001 (3)
O18	0.079 (5)	0.042 (4)	0.022 (3)	−0.031 (4)	0.019 (3)	−0.008 (3)
O19	0.013 (3)	0.033 (3)	0.016 (3)	0.001 (2)	0.003 (2)	−0.005 (2)
O20	0.014 (3)	0.029 (3)	0.025 (3)	−0.002 (2)	0.000 (2)	−0.010 (2)
O1W	0.032 (3)	0.034 (3)	0.014 (3)	0.011 (3)	0.006 (2)	0.003 (3)
O2W	0.048 (4)	0.022 (3)	0.027 (3)	−0.006 (3)	0.005 (3)	−0.002 (2)
O3W	0.015 (3)	0.025 (3)	0.024 (3)	−0.004 (2)	0.004 (2)	−0.006 (2)
O4W	0.019 (3)	0.041 (4)	0.020 (3)	0.009 (3)	0.002 (2)	0.006 (3)
O5W	0.043 (4)	0.028 (3)	0.031 (4)	0.002 (3)	0.005 (3)	−0.005 (3)
O6W	0.041 (4)	0.016 (3)	0.016 (3)	−0.002 (3)	−0.012 (3)	0.003 (2)
O7W	0.024 (3)	0.030 (3)	0.013 (3)	0.003 (2)	0.003 (2)	0.001 (2)

### Geometric parameters (Å, °)

**Table:**

Sm3—O13	2.300 (5)	S3—O19	1.481 (5)
Sm3—O11	2.317 (5)	N1—C5	1.311 (9)
Sm3—O15	2.411 (4)	N1—C2	1.374 (8)
Sm3—O17	2.427 (5)	N2—C5	1.331 (8)
Sm3—O20^i^	2.431 (5)	N2—C3	1.375 (8)
Sm3—O6W	2.432 (5)	N2—H1	0.87 (2)
Sm3—O10	2.484 (4)	N3—C10	1.330 (8)
Sm3—O5W	2.631 (6)	N3—C7	1.384 (8)
Sm3—S3	3.2922 (19)	N4—C10	1.335 (8)
Sm2—O6	2.353 (5)	N4—C8	1.380 (8)
Sm2—O19^i^	2.404 (5)	N4—H2	0.86 (2)
Sm2—O3^ii^	2.408 (5)	C1—O9	1.262 (8)
Sm2—O4W	2.459 (5)	C1—O10	1.272 (7)
Sm2—O3W	2.465 (5)	C1—C2	1.471 (9)
Sm2—O10	2.527 (4)	C2—C3	1.368 (9)
Sm2—O16	2.535 (5)	C3—C4	1.486 (9)
Sm2—O9	2.630 (4)	C4—O11	1.243 (8)
Sm2—O15	2.636 (4)	C4—O12	1.252 (8)
Sm2—C9	2.939 (7)	C5—H5	0.9300
Sm2—C1	2.969 (6)	C6—O13	1.229 (8)
Sm1—O8^iii^	2.327 (5)	C6—O14	1.268 (8)
Sm1—O2^iv^	2.375 (5)	C6—C7	1.496 (9)
Sm1—O5	2.394 (5)	C7—C8	1.382 (9)
Sm1—O2W	2.457 (5)	C8—C9	1.467 (9)
Sm1—O1	2.502 (5)	C9—O16	1.255 (8)
Sm1—O1W	2.515 (5)	C9—O15	1.272 (8)
Sm1—N1	2.552 (6)	C10—H11	0.9300
Sm1—O9	2.553 (4)	O2—Sm1^iv^	2.375 (5)
Sm1—O4	2.699 (5)	O3—Sm2^ii^	2.408 (5)
Sm1—S1	3.2011 (17)	O8—Sm1^i^	2.327 (5)
Co1—O14	2.048 (4)	O19—Sm2^iii^	2.404 (5)
Co1—O14^v^	2.048 (4)	O20—Sm3^iii^	2.431 (5)
Co1—O7W	2.109 (5)	O1W—H1W	0.82 (2)
Co1—O7W^v^	2.109 (5)	O1W—H2W	0.82 (2)
Co1—N3^v^	2.146 (5)	O2W—H3W	0.83 (2)
Co1—N3	2.146 (5)	O2W—H4W	0.82 (2)
S1—O2	1.454 (5)	O3W—H5W	0.83 (2)
S1—O3	1.467 (5)	O3W—H6W	0.83 (2)
S1—O4	1.469 (5)	O4W—H7W	0.81 (2)
S1—O1	1.483 (5)	O4W—H8W	0.81 (2)
S2—O7	1.438 (5)	O5W—H9W	0.82 (2)
S2—O5	1.472 (5)	O5W—H10W	0.81 (2)
S2—O8	1.473 (5)	O6W—H11W	0.81 (2)
S2—O6	1.480 (5)	O6W—H12W	0.80 (2)
S3—O18	1.449 (6)	O7W—H13W	0.81 (2)
S3—O20	1.459 (5)	O7W—H14W	0.81 (2)
S3—O17	1.478 (6)		
			
O13—Sm3—O11	139.58 (16)	O4—Sm1—S1	27.17 (11)
O13—Sm3—O15	74.54 (15)	O14—Co1—O14^v^	180.000 (1)
O11—Sm3—O15	143.87 (16)	O14—Co1—O7W	88.2 (2)
O13—Sm3—O17	120.75 (19)	O14^v^—Co1—O7W	91.8 (2)
O11—Sm3—O17	81.19 (19)	O14—Co1—O7W^v^	91.8 (2)
O15—Sm3—O17	89.23 (17)	O14^v^—Co1—O7W^v^	88.2 (2)
O13—Sm3—O20^i^	85.31 (19)	O7W—Co1—O7W^v^	180.000 (3)
O11—Sm3—O20^i^	93.60 (18)	O14—Co1—N3^v^	101.16 (19)
O15—Sm3—O20^i^	74.62 (16)	O14^v^—Co1—N3^v^	78.84 (19)
O17—Sm3—O20^i^	144.84 (18)	O7W—Co1—N3^v^	88.7 (2)
O13—Sm3—O6W	76.76 (17)	O7W^v^—Co1—N3^v^	91.3 (2)
O11—Sm3—O6W	77.13 (18)	O14—Co1—N3	78.84 (19)
O15—Sm3—O6W	133.98 (17)	O14^v^—Co1—N3	101.16 (19)
O17—Sm3—O6W	75.58 (19)	O7W—Co1—N3	91.3 (2)
O20^i^—Sm3—O6W	137.37 (18)	O7W^v^—Co1—N3	88.7 (2)
O13—Sm3—O10	141.54 (15)	N3^v^—Co1—N3	180.000 (2)
O11—Sm3—O10	74.32 (16)	O2—S1—O3	109.0 (3)
O15—Sm3—O10	69.57 (15)	O2—S1—O4	111.9 (3)
O17—Sm3—O10	72.68 (17)	O3—S1—O4	111.1 (3)
O20^i^—Sm3—O10	72.44 (16)	O2—S1—O1	110.0 (3)
O6W—Sm3—O10	139.86 (17)	O3—S1—O1	108.9 (3)
O13—Sm3—O5W	69.51 (18)	O4—S1—O1	105.9 (3)
O11—Sm3—O5W	73.27 (18)	O2—S1—Sm1	120.5 (2)
O15—Sm3—O5W	127.62 (17)	O3—S1—Sm1	130.2 (2)
O17—Sm3—O5W	142.04 (19)	O4—S1—Sm1	57.0 (2)
O20^i^—Sm3—O5W	65.80 (18)	O1—S1—Sm1	49.36 (19)
O6W—Sm3—O5W	71.7 (2)	O7—S2—O5	110.8 (3)
O10—Sm3—O5W	124.27 (17)	O7—S2—O8	111.8 (3)
O13—Sm3—S3	96.29 (14)	O5—S2—O8	107.8 (3)
O11—Sm3—S3	101.50 (14)	O7—S2—O6	111.0 (4)
O15—Sm3—S3	80.81 (12)	O5—S2—O6	108.4 (3)
O17—Sm3—S3	24.47 (14)	O8—S2—O6	106.9 (3)
O20^i^—Sm3—S3	154.02 (12)	O18—S3—O20	112.5 (4)
O6W—Sm3—S3	67.43 (14)	O18—S3—O17	107.1 (4)
O10—Sm3—S3	91.30 (11)	O20—S3—O17	109.2 (3)
O5W—Sm3—S3	138.88 (14)	O18—S3—O19	109.7 (3)
O6—Sm2—O19^i^	77.78 (18)	O20—S3—O19	109.8 (3)
O6—Sm2—O3^ii^	73.35 (18)	O17—S3—O19	108.5 (3)
O19^i^—Sm2—O3^ii^	75.41 (16)	O18—S3—Sm3	64.5 (3)
O6—Sm2—O4W	134.97 (18)	O20—S3—Sm3	120.6 (2)
O19^i^—Sm2—O4W	139.39 (17)	O17—S3—Sm3	42.9 (2)
O3^ii^—Sm2—O4W	129.63 (17)	O19—S3—Sm3	127.5 (2)
O6—Sm2—O3W	90.2 (2)	C5—N1—C2	105.9 (6)
O19^i^—Sm2—O3W	148.19 (16)	C5—N1—Sm1	133.2 (4)
O3^ii^—Sm2—O3W	72.93 (16)	C2—N1—Sm1	120.6 (4)
O4W—Sm2—O3W	67.74 (17)	C5—N2—C3	107.9 (6)
O6—Sm2—O10	88.95 (18)	C5—N2—H1	120 (6)
O19^i^—Sm2—O10	81.35 (15)	C3—N2—H1	131 (6)
O3^ii^—Sm2—O10	153.19 (15)	C10—N3—C7	104.9 (6)
O4W—Sm2—O10	77.07 (17)	C10—N3—Co1	144.9 (5)
O3W—Sm2—O10	128.31 (15)	C7—N3—Co1	110.2 (4)
O6—Sm2—O16	140.10 (17)	C10—N4—C8	108.7 (5)
O19^i^—Sm2—O16	75.70 (17)	C10—N4—H2	123 (6)
O3^ii^—Sm2—O16	71.47 (17)	C8—N4—H2	126 (5)
O4W—Sm2—O16	83.19 (18)	O9—C1—O10	119.7 (6)
O3W—Sm2—O16	96.93 (16)	O9—C1—C2	117.5 (6)
O10—Sm2—O16	115.56 (14)	O10—C1—C2	122.9 (6)
O6—Sm2—O9	70.15 (16)	O9—C1—Sm2	62.2 (3)
O19^i^—Sm2—O9	120.47 (16)	O10—C1—Sm2	57.6 (3)
O3^ii^—Sm2—O9	134.74 (16)	C2—C1—Sm2	175.1 (5)
O4W—Sm2—O9	68.06 (17)	C3—C2—N1	109.4 (6)
O3W—Sm2—O9	81.28 (15)	C3—C2—C1	135.0 (6)
O10—Sm2—O9	50.23 (14)	N1—C2—C1	115.4 (6)
O16—Sm2—O9	149.71 (16)	C2—C3—N2	105.2 (6)
O6—Sm2—O15	140.23 (18)	C2—C3—C4	135.0 (6)
O19^i^—Sm2—O15	69.01 (16)	N2—C3—C4	119.8 (6)
O3^ii^—Sm2—O15	116.59 (15)	O11—C4—O12	124.0 (7)
O4W—Sm2—O15	70.80 (16)	O11—C4—C3	119.9 (6)
O3W—Sm2—O15	129.46 (16)	O12—C4—C3	116.1 (6)
O10—Sm2—O15	65.45 (14)	N1—C5—N2	111.6 (6)
O16—Sm2—O15	50.12 (14)	N1—C5—H5	124.2
O9—Sm2—O15	108.56 (14)	N2—C5—H5	124.2
O6—Sm2—C9	143.70 (19)	O13—C6—O14	123.2 (6)
O19^i^—Sm2—C9	66.30 (18)	O13—C6—C7	120.7 (6)
O3^ii^—Sm2—C9	92.08 (18)	O14—C6—C7	116.0 (6)
O4W—Sm2—C9	79.88 (19)	C8—C7—N3	110.4 (6)
O3W—Sm2—C9	117.63 (18)	C8—C7—C6	133.2 (6)
O10—Sm2—C9	90.65 (17)	N3—C7—C6	116.1 (6)
O16—Sm2—C9	25.15 (17)	N4—C8—C7	104.3 (5)
O9—Sm2—C9	133.08 (17)	N4—C8—C9	119.9 (6)
O15—Sm2—C9	25.64 (16)	C7—C8—C9	135.7 (6)
O6—Sm2—C1	77.54 (18)	O16—C9—O15	120.3 (6)
O19^i^—Sm2—C1	100.65 (17)	O16—C9—C8	117.9 (6)
O3^ii^—Sm2—C1	150.81 (18)	O15—C9—C8	121.6 (6)
O4W—Sm2—C1	71.87 (18)	O16—C9—Sm2	59.1 (3)
O3W—Sm2—C1	105.37 (17)	O15—C9—Sm2	63.8 (3)
O10—Sm2—C1	25.15 (15)	C8—C9—Sm2	159.8 (5)
O16—Sm2—C1	136.44 (17)	N3—C10—N4	111.7 (6)
O9—Sm2—C1	25.14 (15)	N3—C10—H11	124.2
O15—Sm2—C1	87.51 (16)	N4—C10—H11	124.2
C9—Sm2—C1	113.14 (19)	S1—O1—Sm1	103.9 (2)
O8^iii^—Sm1—O2^iv^	82.97 (18)	S1—O2—Sm1^iv^	157.7 (3)
O8^iii^—Sm1—O5	132.72 (18)	S1—O3—Sm2^ii^	151.1 (3)
O2^iv^—Sm1—O5	143.93 (18)	S1—O4—Sm1	95.8 (2)
O8^iii^—Sm1—O2W	74.9 (2)	S2—O5—Sm1	135.0 (3)
O2^iv^—Sm1—O2W	134.42 (18)	S2—O6—Sm2	141.8 (3)
O5—Sm1—O2W	71.7 (2)	S2—O8—Sm1^i^	154.6 (3)
O8^iii^—Sm1—O1	132.36 (16)	C1—O9—Sm1	123.4 (4)
O2^iv^—Sm1—O1	84.65 (16)	C1—O9—Sm2	92.6 (4)
O5—Sm1—O1	74.15 (16)	Sm1—O9—Sm2	141.08 (19)
O2W—Sm1—O1	81.88 (18)	C1—O10—Sm3	141.3 (4)
O8^iii^—Sm1—O1W	149.16 (18)	C1—O10—Sm2	97.2 (4)
O2^iv^—Sm1—O1W	73.88 (18)	Sm3—O10—Sm2	112.87 (16)
O5—Sm1—O1W	70.95 (18)	C4—O11—Sm3	157.0 (5)
O2W—Sm1—O1W	135.9 (2)	C6—O13—Sm3	155.7 (5)
O1—Sm1—O1W	66.14 (16)	C6—O14—Co1	118.2 (4)
O8^iii^—Sm1—N1	82.36 (18)	C9—O15—Sm3	152.2 (4)
O2^iv^—Sm1—N1	72.20 (17)	C9—O15—Sm2	90.6 (4)
O5—Sm1—N1	103.37 (18)	Sm3—O15—Sm2	111.56 (17)
O2W—Sm1—N1	140.42 (19)	C9—O16—Sm2	95.7 (4)
O1—Sm1—N1	135.86 (17)	S3—O17—Sm3	112.7 (3)
O1W—Sm1—N1	71.45 (18)	S3—O19—Sm2^iii^	142.2 (3)
O8^iii^—Sm1—O9	73.54 (17)	S3—O20—Sm3^iii^	146.5 (3)
O2^iv^—Sm1—O9	130.86 (16)	Sm1—O1W—H1W	112 (6)
O5—Sm1—O9	68.57 (16)	Sm1—O1W—H2W	111 (6)
O2W—Sm1—O9	80.04 (17)	H1W—O1W—H2W	104 (3)
O1—Sm1—O9	142.05 (15)	Sm1—O2W—H3W	125 (5)
O1W—Sm1—O9	106.70 (16)	Sm1—O2W—H4W	133 (5)
N1—Sm1—O9	62.47 (16)	H3W—O2W—H4W	101 (7)
O8^iii^—Sm1—O4	78.70 (16)	Sm2—O3W—H5W	118 (6)
O2^iv^—Sm1—O4	72.14 (16)	Sm2—O3W—H6W	114 (5)
O5—Sm1—O4	114.36 (16)	H5W—O3W—H6W	102 (3)
O2W—Sm1—O4	64.80 (17)	Sm2—O4W—H7W	116 (6)
O1—Sm1—O4	53.73 (15)	Sm2—O4W—H8W	123 (6)
O1W—Sm1—O4	112.03 (16)	H7W—O4W—H8W	106 (7)
N1—Sm1—O4	141.24 (17)	Sm3—O5W—H9W	94 (6)
O9—Sm1—O4	139.76 (15)	Sm3—O5W—H10W	139 (7)
O8^iii^—Sm1—S1	105.85 (13)	H9W—O5W—H10W	105 (8)
O2^iv^—Sm1—S1	74.94 (12)	Sm3—O6W—H11W	120 (5)
O5—Sm1—S1	95.75 (12)	Sm3—O6W—H12W	129 (5)
O2W—Sm1—S1	73.64 (14)	H11W—O6W—H12W	107 (3)
O1—Sm1—S1	26.73 (11)	Co1—O7W—H13W	104 (6)
O1W—Sm1—S1	87.74 (12)	Co1—O7W—H14W	116 (6)
N1—Sm1—S1	144.82 (13)	H13W—O7W—H14W	105 (3)
O9—Sm1—S1	152.67 (11)		

Symmetry codes: (i) *x*−1, *y*, *z*; (ii) −*x*, −*y*, −*z*+1; (iii) *x*+1, *y*, *z*; (iv) −*x*+1, −*y*+1, −*z*+1; (v) −*x*, −*y*−1, −*z*+2.

### Hydrogen-bond geometry (Å, °)

**Table:**

*D*—H···*A*	*D*—H	H···*A*	*D*···*A*	*D*—H···*A*
N2—H1···O18^vi^	0.87 (6)	2.06 (5)	2.916 (8)	170 (6)
O1W—H1W···O7	0.82 (5)	2.52 (8)	3.140 (8)	133 (7)
O1W—H1W···O16^vi^	0.82 (5)	2.12 (6)	2.777 (6)	136 (8)
N4—H2···O7^vii^	0.87 (4)	1.94 (5)	2.803 (8)	171 (8)
O1W—H2W···O1^viii^	0.82 (5)	2.51 (6)	3.247 (7)	150 (7)
O2W—H3W···O3W	0.83 (5)	2.02 (5)	2.809 (8)	160 (6)
O2W—H4W···O6^ii^	0.83 (4)	2.55 (4)	3.303 (9)	152 (8)
O2W—H4W···O8^ii^	0.83 (4)	2.53 (7)	3.204 (8)	140 (6)
O3W—H5W···O4^ix^	0.83 (4)	1.98 (5)	2.789 (7)	166 (8)
O3W—H6W···O1^ii^	0.83 (5)	1.93 (5)	2.732 (7)	161 (5)
O4W—H7W···O17	0.81 (5)	2.59 (6)	3.270 (8)	142 (7)
O4W—H7W···O19	0.81 (5)	2.20 (6)	2.874 (7)	141 (5)
O4W—H8W···O6^iii^	0.81 (7)	2.47 (7)	3.186 (8)	149 (6)
O5W—H10W···O7W^x^	0.82 (6)	2.10 (6)	2.794 (8)	142 (6)
O6W—H11W···O14^xi^	0.81 (4)	1.99 (5)	2.751 (7)	157 (5)
O6W—H12W···O5W^xi^	0.81 (6)	2.27 (7)	3.053 (8)	162 (7)
O7W—H13W···O11^x^	0.81 (5)	2.45 (5)	3.063 (6)	133 (7)
O7W—H13W···O12^x^	0.81 (5)	2.06 (6)	2.855 (7)	168 (7)
O7W—H14W···O12^xii^	0.81 (5)	1.85 (5)	2.661 (7)	174 (7)
C5—H5···O3^iv^	0.93	2.52	3.388 (8)	155.
C10—H11···O17^xii^	0.93	2.54	3.430 (9)	161.

Symmetry codes: (vi) *x*, *y*+1, *z*; (vii) *x*, *y*−1, *z*; (viii) −*x*, −*y*+1, −*z*+1; (ii) −*x*, −*y*, −*z*+1; (ix) −*x*+1, −*y*, −*z*+1; (iii) *x*+1, *y*, *z*; (x) −*x*, −*y*, −*z*+2; (xi) −*x*+1, −*y*, −*z*+2; (xii) *x*−1, *y*−1, *z*; (iv) −*x*+1, −*y*+1, −*z*+1.
